# Impact of glaucoma on outcomes after epiretinal membrane surgery. a pairwise and post-hoc single-arm meta-analysis

**DOI:** 10.1007/s00417-026-07188-2

**Published:** 2026-03-26

**Authors:** Raphaela Masetto C. Fuganti, Tiago Nelson O. Rassi, Maikon V. Fuganti, Neeran Narainswami, Jorge Rocha, Sunir Garg, Mauricio Maia, Antonio Marcelo B. Casella, Raphaela Masetto C. Fuganti, Antonio Marcelo B. Casela

**Affiliations:** 1 State University of Londrina, Londrina, Brazil; 2 Federal University of São Paulo, São Paulo, Brazil; 3 UNIMED North of Paraná, Cornélio Procópio, Brazil; 4 University of Cape Town, Red Cross Hospital, Rondebosch, Cape Town, South Africa; 5 iRetina Eye Institute, Salvador, Brazil; 6 Wills Eye Hospital, Philadelphia, United States of America

**Keywords:** Epiretinal membrane, Glaucoma, Vitrectomy, ILM peeling, Visual acuity, Meta-analysis, Visual field

## Abstract

**Purpose:**

To evaluate whether glaucoma modifies functional, anatomical, and intraocular pressure outcomes after pars plana vitrectomy (PPV) with internal limiting membrane (ILM) peeling for idiopathic epiretinal membrane (ERM).

**Methods:**

PubMed, Embase, and Cochrane Library were searched through April 27, 2025, for comparative studies of PPV with ILM peeling in glaucomatous versus non-glaucomatous eyes. Primary outcome was the change (Δ) in best-corrected visual acuity (BCVA); secondary outcomes were changes (Δ) in central macular thickness (CMT), intraocular pressure (IOP), and number of glaucoma medications. Risk of bias was assessed with ROBINS-I and certainty of evidence with GRADE. A post-hoc single-arm synthesis summarized outcomes in glaucomatous eyes. PROSPERO registration: CRD420251041680.

**Results:**

Six studies (779 eyes: 256 glaucomatous, 523 non-glaucomatous) contributed to the pairwise meta-analysis, and three additional studies (325 glaucomatous eyes) informed single-arm data. Non-glaucomatous eyes achieved greater BCVA improvement (mean difference − 6.95 ETDRS letters; 95% CI − 11.54 to − 2.35; *p* < 0.01) and larger CMT reduction after outlier removal (+ 39.50 μm; 95% CI 11.48 to 67.53; *p* = 0.006). Glaucomatous eyes still improved significantly (BCVA + 10.03 ETDRS letters; CMT − 102.05 μm), with stable IOP (Δ + 0.64 mmHg; *p* = 0.12) but increased medication use (+ 0.61 drops; *p* < 0.001). Follow-up ranged from 6 to 12 months, and overall evidence certainty was high.

**Conclusions:**

Glaucoma attenuates, but does not eliminate, the visual and anatomical benefits of ERM surgery. However, central visual field safety cannot be guaranteed, particularly in moderate to advanced disease. Careful postoperative glaucoma monitoring and individualized management are recommended.

**Supplementary Information:**

The online version contains supplementary material available at 10.1007/s00417-026-07188-2.

## Key messages

*****What was known before***:**

- Glaucomatous eyes may show reduced functional recovery after epiretinal membrane (ERM) surgery due to inner retinal damage, but previous studies were small and inconsistent.

*****What this study adds***:**

- This meta-analysis demonstrate that glaucomatous eyes achieve smaller visual gains than non-glaucomatous eyes after ERM surgery (-6.95 ETDRS letters), yet still experience clinically relevant improvements in vision (+10.03 ETDRS letters) and macular thickness (m). Intraocular pressure (IOP) remains stable, although additional glaucoma medication may be required.

**Supplementary Information:**

The online version contains supplementary material available at 10.1007/s00417-026-07188-2.

## Introduction

Idiopathic epiretinal membrane (ERM) is a common macular condition characterized by fibrocellular proliferation on the inner retinal surface, often leading to visual distortion and progressive visual acuity loss [1–8]. Pars plana vitrectomy (PPV) with or without internal limiting membrane (ILM) peeling is widely regarded as the standard surgical treatment for symptomatic ERM, promoting both anatomical restoration and functional improvement in most patients [9].

Glaucoma, a chronic optic neuropathy associated with retinal ganglion cell loss and thinning of the inner retinal layers, may influence visual prognosis after ERM surgery. Although some recent studies have investigated this association, the existing evidence remains limited and heterogeneous. Questions persist regarding whether patients with glaucoma derive the same degree of visual and anatomical benefit from surgery as those without glaucoma, and whether the presence of glaucoma increases the risk of postoperative complications or structural damage progression.[1–9].

This systematic review and meta-analysis aimed to evaluate both functional and structural outcomes following vitrectomy in patients with ERM, comparing those with and without a confirmed diagnosis of glaucoma. By addressing this distinction, the study seeks to generate clinically meaningful evidence to inform surgical decision-making and patient counseling in the context of coexisting ERM and glaucoma.

## Methods

### Eligibility criteria

This systematic review and meta-analysis were performed and reported in accordance with the Cochrane Collaboration Handbook for Systematic Review of Interventions and the Preferred Reporting Items for Systematic Reviews and Meta-Analysis (PRISMA) Statement guidelines [10, 11].

Inclusion criteria were restricted to studies that met all the following: (1) comparative design, including randomized controlled trials, prospective or retrospective cohort studies, or case-control studies; (2) patients with a confirmed diagnosis of glaucoma (any type) and idiopathic epiretinal membrane (ERM), who underwent PPV with internal limiting membrane (ILM) peeling; (3) control group consisting of patients without glaucoma, also with idiopathic ERM, treated with the same surgical technique (vitrectomy with ILM peeling); (4) reporting of at least one of the following outcomes: best-corrected visual acuity (BCVA) improvement, visual field progression, structural changes detected by optical coherence tomography (e.g., retinal nerve fiber layer thickness, ganglion cell complex thickness), or glaucoma-related complications (e.g., intraocular pressure (IOP) elevation, glaucoma progression, or need for additional treatment); (5) articles published in English; (6) minimum postoperative follow-up of 3 months; and (7) availability of extractable quantitative data suitable for meta-analysis or descriptive comparison.

Studies were excluded if they lacked clear glaucoma diagnostic criteria, employed different surgical techniques between groups, or were case reports, narrative reviews, editorials, letters, or conference abstracts without complete data. In cases of duplicate or overlapping data, the most complete or recent publication was included.

### Search strategy and data extraction

A comprehensive literature search was conducted across PubMed, Embase, and the Cochrane Library, from database inception through April 27, 2025. The search strategy included the following terms and their synonyms: (“epiretinal membrane” OR “macular pucker”) AND (“vitrectomy” OR “pars plana vitrectomy”) AND (“internal limiting membrane peeling”) AND (“glaucoma”) (Supplemental Material 1). No restrictions were applied regarding publication date or language. All retrieved references were managed using Zotero, which facilitated deduplication and organization of citations.

Additionally, the reference lists of all included articles and prior systematic reviews and meta-analyses were manually screened to identify any additional eligible studies. Two independent reviewers (RMF and MVF) screened the titles and abstracts using Zotero, followed by full-text assessment according to predefined eligibility criteria. Data extraction and quality assessment were independently performed by the same reviewers (RMF and MVF), using a standardized data collection form. Any discrepancies were resolved through discussion or consultation with a third reviewer (TNR).

The protocol for this meta-analysis was prospectively registered in PROSPERO (CRD420251041680) on April 27, 2025. All prespecified outcomes were considered.

Visual field (VF) progression, retinal nerve fiber layer (RNFL) thickness, and ganglion cell complex (GCC) thinning were prespecified secondary outcomes in the PROSPERO protocol. However, these endpoints were not eligible for quantitative synthesis because the included studies used heterogeneous VF indices, variable definitions of progression, nonuniform OCT devices and segmentation algorithms, or inconsistent follow-up timepoints, with insufficient extractable data to compute effect sizes. Therefore, these outcomes were summarized narratively and interpreted as supportive, hypothesis-generating evidence only.

### Endpoints and sensitivity analyses

The outcomes analyzed in this meta-analysis (Table 1) were: (1) change in BCVA in ETDRS (Early Treatment Diabetic Retinopathy Study) letters; (2) change in IOP in mmHg; and (3) change in central macular thickness (CMT) in micrometers (µm). The difference between postoperative and preoperative values (Δ) was calculated by subtracting final follow-up from the baseline. When final values were not explicitly reported but the mean change (Δ) was graphically presented with error bars, data were extracted using the WebPlotDigitizer tool. [12] It was assumed that the error bars represent 95% confidence intervals. Standard deviations (SD) were then estimated following guidance from the Cochrane Handbook for Systematic Reviews of Interventions [10] on converting confidence intervals into standard deviations. In the absence of directly reported final SD, a conservative (*r* = 0,5) estimate was derived based on the combined variance of baseline and change scores, in accordance with established approaches for handling incomplete outcome data in systematic reviews. When multiple timepoints were available, we extracted the measurement closest to 12 months.

**Table 1:** Main characteristics of included studies

Pairwise					Glaucoma					Without Glaucoma		
Study	Type	Country	FU	S (*n*)	Male (%, *n*)	Age, Mean (SD)	Severity	Glaucoma stage	ERM stage	Male (%, *n*)	Age, Mean (SD)	ERM stage
Ko et al., 2022 [2]	Retrospective cohort	Taiwan	12	7	32.3% (10/31)	68.55 (8.31)	Most severe	MD: −10,2 (7,1)	1:0/31 2:3/31 3:20/31 4:8/31	65.9% (172/261)	69.9 (8.0)	NA
Higashide et al., 2025 [1]	Prospective cohort	Japan	12	1	40.4% (19/47)	65.6 (7.3)	Mild to severe	MD: −9.33 (6.79)	1:8/47 2:13/47 3:20/47 4:6/47	34.8% (16/46)	64.0 (6.8)	1: 4/46 2: 10/46 3: 25/46 4: 7/46
Peck et al., 2022 [5]	Retrospective case-control	USA	6	NA	44.7% (46/103)	71.6 (7.6)	Mild to severe	Mild: 33/103; moderate: 31/103; severe 39/103	1:9/103 2:27/103 3:47/103 4:20/103	48.2% (67/139)	73.2 (6.1)	1: 9/139 2:29/139 3:57/139 4:44/139
Lyssek-Boron et al., 2017 [9]	Retrospective cohort	Poland	12	1	57.1% (12/21)	69.6 (7.6)	NA (severe glaucoma excluded)	NA	NA	36.8% (7/19)	70.9 (6.7)	NA
Govetto et al., 2017 [3]	Retrospective cohort	USA	6	1	NA	NA	Mild to severe	mild: 9/20; moderate: 1/20 severe 10/20	1:3/20 2:10/20 3:4/20 4:3/20	NA	NA	1: 0/24 2:5/24 3:15/24 4: 4/24
Han et al., 2025 [4]	Retrospective cohort	South Korea	6	1	50.0% (17/34)	69.7 (8.0)	Moderate	MD: −8.26 (5.54)	NA	50% (17/34)	65.8 (7.7)	NA
**Post-hoc Single-arm**												
Yoshida et al., 2019 [8]	Retrospective cohort	Japan	6	1	30.0% (6/20)	68,9 (8,1)	Moderate	MD: −8,7 (NA)	NA			
Kanai et al., 2024 [6]	Retrospective cohort	Japan	6	NA (> 1)	55.0% (17/31)	76.8 (8,7)	Moderate	MD: −7,3 (5,5)	1:6/31; 2:10/31; 3:10/31; 4:5/31			
Kaneko et al., 2021 [7]	Retrospective cohort	Japan	6	2	61.0% (11/18)	68.3 (7,2)	NA	NA	NA			

ERM. Post-hoc single-arm studies report outcomes exclusively in glaucomatous eyes. BCVA = Best-Corrected Visual Acuity; CMT = Central Macular Thickness; ERM = Epiretinal Membrane; FU = Follow-up in months; ILM = Internal Limiting Membrane; IOP = Intraocular Pressure; MD = Mean Deviation (Visual Field, dB); NA = Not Available; SD = Standard Deviation; S = Number of surgeons

In addition of these primary outcomes, safety outcomes, including intraoperative or postoperative adverse events and glaucoma-related complications (e.g., disease progression or need for intensified treatment) were prespecified in the PROSPERO protocol and were extracted when available. Other secondary outcomes, such as visual field progression, RNFL and GCC thinning, were also considered during eligibility screening. However, these were not consistently reported across the included studies and could not be meta-analyzed. When available, findings were narratively summarized.

### Quality Assessment

The risk of bias for observational studies was evaluated using the Risk of Bias in Non-Randomized Studies - of interventions tool (ROBINS-I) [13] (Supplemental Fig. 3). Two independent reviewers (RMF and MVF) assessed the risk of bias, with discrepancies resolved through discussion and mutual consensus. The certainty of evidence for each outcome was rated using the Grading of Recommendations, Assessment, Development, and Evaluation (GRADE) [14, 15] framework (Supplemental Fig. 4). The following domains were evaluated: risk of bias, inconsistency, indirectness, imprecision, and publication bias. Two reviewers (RMF and MVF) independently performed GRADE assessments. Any disagreements were resolved by consensus or, if necessary, with a third reviewer (TNR). Summary of Findings table were generated with GRADEpro GDT [14, 15].

### Statistical analysis

Meta-analyses were performed using a random-effects model to account for anticipated clinical and methodological heterogeneity. For continuous outcomes, which were pooled using mean differences and corresponding 95% confidence intervals (CI).

Heterogeneity was assessed using the I2 statistic and Cochrane Q test; p-values < 0.10 and I2 > 25% were considered significant for heterogeneity. Sensitivity analyses were conducted using a leave-one-out approach, whereby each study was sequentially excluded to evaluate the robustness of the overall estimates.

Assessment of publication bias through funnel plots or formal statistical tests was not performed, as fewer than 10 studies were available.

All statistical analyses were performed using the meta package within RStudio[16].

### Post-hoc single-arm meta-analysis

In addition, a post-hoc single-arm meta-analysis was conducted to explore visual and structural outcomes exclusively in eyes with glaucoma undergoing vitrectomy with ILM peeling. This analysis was performed to provide additional insight into within-group changes and the benefit of surgery in glaucomatous eyes. The decision to perform this analysis arose from the observation that several included studies provided detailed baseline and postoperative outcomes for glaucomatous eyes but lacked comparable control data, rendering them ineligible for pairwise synthesis. Rather than exclude these data, we elected to incorporate them into a structured single-arm framework, thereby maximizing information use and increasing the precision of within-group effect estimates.

The outcomes evaluated in the single-arm analysis included: (1) change in BCVA (in ETDRS letters); (2) change in CMT (in µm); (3) change in IOP (in mmHg); (4) change in the number of topical glaucoma medications. Mean differences were calculated as the change from final to baseline to the last follow-up visit. Random-effects models were applied throughout. Heterogeneity was assessed using I2 and Cochrane Q test, and sensitivity analyses (leave-one-out, meta-regression, and subgroup analysis) were performed. This post-hoc approach enriched the analysis by enabling the evaluation of trends and outcome consistency within the glaucoma group, especially for clinically relevant variables that could not be assessed in comparative analyses due to data limitations or design inconsistencies among included studies.

## Results

### Study selection and baseline characteristics

The initial search yielded 771 results. After the removal of duplicate records and ineligible studies, 18 remained and were fully reviewed based on the inclusion criteria. Of these, 6 studies [1–5, 9] were included in the final comparative analysis, comprising a total of 256 glaucomatous eyes (from 4 retrospective cohort studies, 1 prospective cohort, and 1 retrospective case-control study). In addition, 3 studies [6–8] that lacked appropriate control groups but provided relevant data on glaucomatous eyes were included in a post-hoc single-arm meta-analysis, bringing 325 eyes evaluated (Table 1). One study from overlapping [1, 20, 23] was included in the quantitative analyzes, while the others were narratively summarized. Prespecified neurofunctional outcomes (VF progression) and neuroretinal OCT metrics (RNFL and GCC) were not meta-analyzed and were therefore reported narratively in a dedicated section below, given the heterogeneity and insufficient standardized data.

Across studies, patients with glaucoma were generally older, with a mean age ranging from 65.6 to 71.6 years, and predominantly male in 32,3–57,1% of cases. Most included studies [1, 3, 5, 6, 8] (62.5%) evaluated patients with mild to moderate glaucoma. Two studies [6, 8] focused exclusively on moderate cases, and one study [2] predominantly included severe glaucoma. ERM staging varied across studies but was generally balanced between groups. Surgical techniques, including the number of operating surgeons and use of dyes, differed substantially. Primary outcomes (BCVA, CMT, IOP) were assessed at 6 to 12 months postoperatively.

Reporting on lens status and glaucoma subtype were inconsistent, with most studies pooling phakic and pseudophakic eyes as well as different glaucoma types without stratified analyses.

## Pooled analyses

### BCVA

**Fig. 1:**
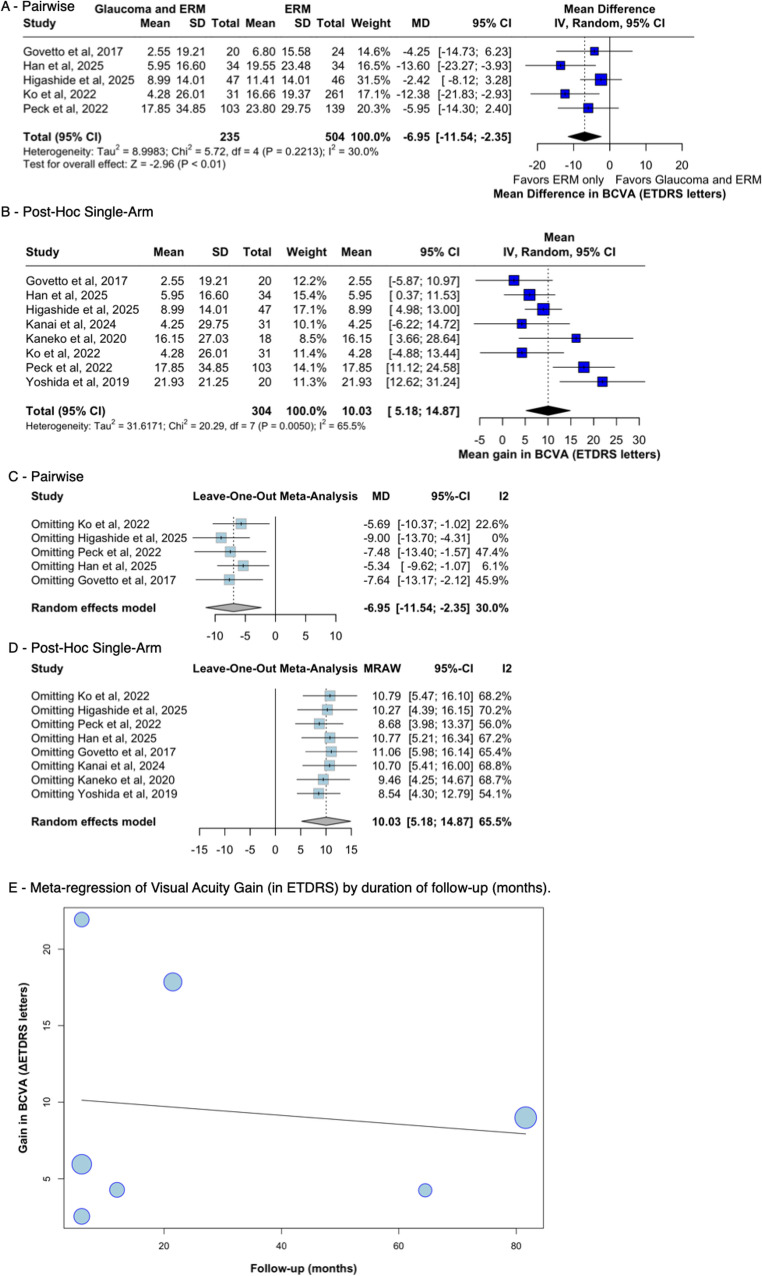
Visual outcomes - forest plots summarizing mean visual acuity gain (ETDRS letters) and sensitivity analysis and meta-regression of best-corrected visual acuity (BCVA) gain (ETDRS letters) after vitrectomy with ILM peeling in glaucomatous eyes (A) Pairwise meta-analysis comparing glaucomatous versus non-glaucomatous eyes. Glaucomatous eyes showed significantly less visual gain (mean difference = − 6.95 ETDRS letters; 95% CI: −11.54 to − 2.35; *p* < 0.01; I2 = 30.0%) (B) Post-hoc single-arm meta-analysis. Greater visual improvement was observed when peeling was consistently performed (mean = 10.77 ETDRS letters; 95% CI: 5.21 to 16.34). In contrast, a single study with variable ILM peeling showed a gain of + 5.95 ETDRS letters (95% CI: 0.37 to 11.53). The difference between subgroups was not statistically significant (*p* = 0.23) (C) Leave-one-out sensitivity analysis for the pairwise meta-analysis. The direction and significance of the effect remained stable across exclusions (pooled MD = − 6.95 ETDRS letters; 95% CI: −11.54 to − 2.35; I2 = 30.0%), confirming the robustness of the between-group difference (D) Leave-one-out sensitivity analysis for the single-arm meta-analysis. Visual gains remained consistent (pooled mean = + 10.03 ETDRS letters; 95% CI: 5.18 to 14.87; I2 = 65.5%) regardless of individual study exclusion (E) Meta-regression assessing the association between follow-up duration (in months) and BCVA gain. No significant correlation was observed, suggesting visual improvements were not strongly dependent on follow-up length

Pooled analysis indicated that glaucomatous eyes experienced significantly less improvement in BCVA after ERM surgery compared with non-glaucomatous controls (mean difference: −6.95 ETDRS letters; 95% CI: −11.54 to − 2.35; *p* < 0.01; I2 = 30.0%) (Fig. 1A). Nonetheless, a post hoc single-arm meta-analysis confirmed that eyes with glaucoma still achieved a clinically meaningful visual gain postoperatively (pooled mean gain: +10.03 ETDRS letters; 95% CI: 5.18 to 14.87; I2 = 65.5%) (Fig. 1B).

Leave-one-out sensitivity analysis of both pairwise and post-hoc single-arm meta-analysis reinforced our findings (Fig. 1C e 1D).

In subgroup analysis by ILM peeling in the single arm post-hoc, eyes that underwent peeling showed a pooled gain of + 10.77 letters (95% CI: 5.21 to 16.34; I2 = 67.2%), whereas a single study with variable peeling reported a lower gain (+ 5.95 letters; 95% CI: 0.37 to 11.53; I2 = 0.0%). The between-subgroup difference was not statistically significant (*p* = 0.23) (Supplemental Fig. 5A). No significant subgroup effect was observed between dye-assisted and non-dye-assisted studies (Supplemental Fig. 5B).

Meta-regression of the post-hoc single arm analysis including seven studies showed no significant association between follow-up duration and BCVA gain (β = − 0.029 letters/month; 95% CI: − 0.22 to 0.16; *p* = 0.77; R2 = 0%; I2 = 74.8%) (Fig. 1E). Residual heterogeneity remained high and significant (QE = 19.12; *p* = 0.0018) indicating that follow-up duration did not account for the variability observed across studies.

### CMT

**Fig. 2:**
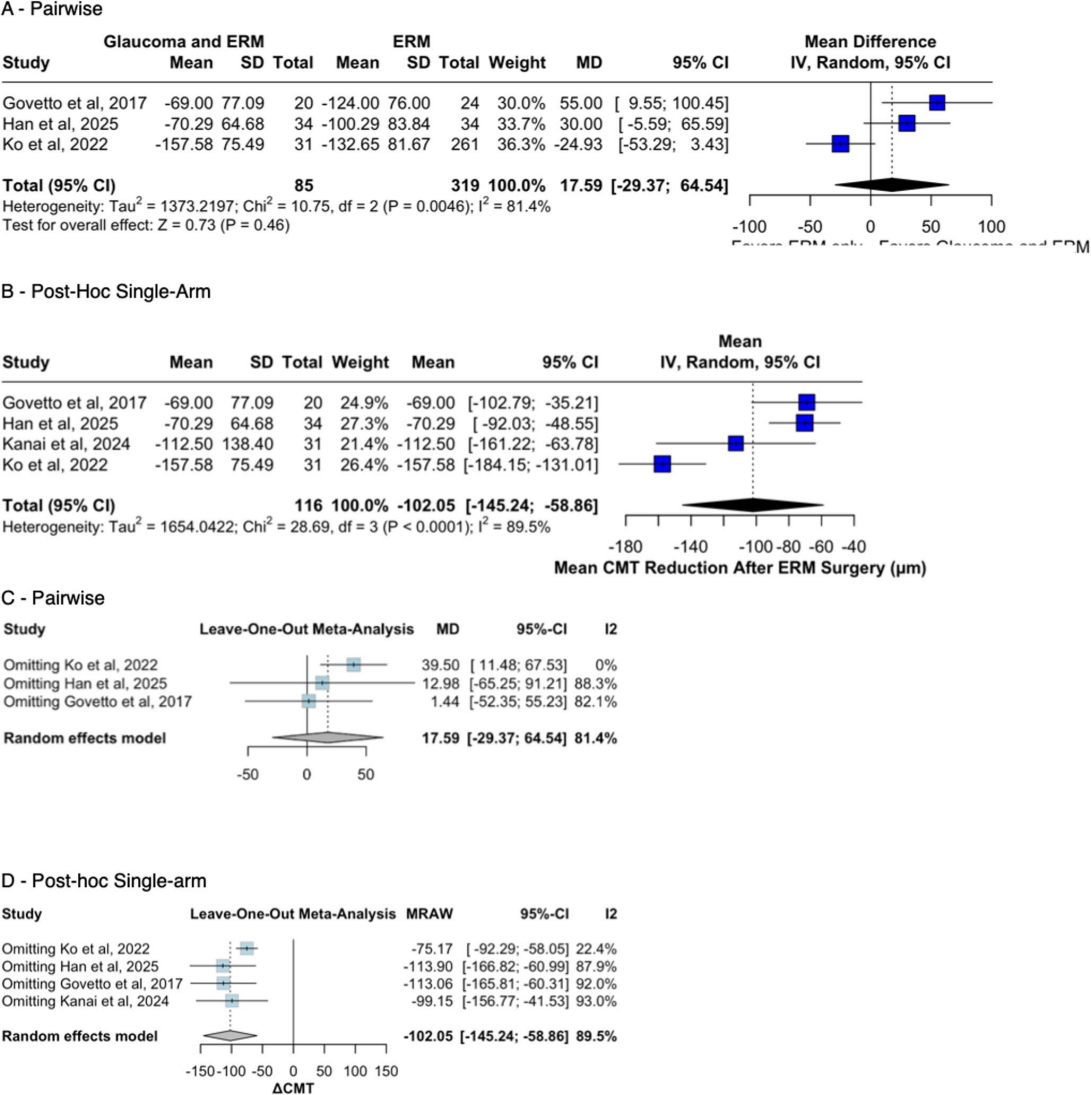
Anatomical outcomes - forest plots summarizing central macular thickness (CMT) reduction and sensitivity analysis after vitrectomy for epiretinal membrane (ERM) in glaucomatous eyes. (**A**) Pairwise meta-analysis comparing glaucomatous versus non-glaucomatous eyes. The pooled mean difference in CMT change was not statistically significant (mean difference = 17.59 μm; 95% CI: −29.37 to 64.54; p = 0.46; I2 = 81.4%), indicating similar anatomical response between groups. (**B**) Post-hoc single-arm meta-analysis pooling glaucomatous eyes only (n = 116). The overall mean CMT reduction was significant (− 102.05 μm; 95% CI: −145.24 to − 58.86; p < 0.0001; I2 = 89.5%), confirming a robust anatomical response after ERM surgery in this subgroup. (**C**) Leave-one-out sensitivity analysis of the pairwise meta-analysis comparing glaucomatous and non-glaucomatous eyes. The pooled mean difference remained non-significant (MD = + 17.59 μm; 95% CI: −29.37 to 64.54; I2 = 81.4%) across exclusions, indicating that results were not driven by any single study. (**D**) Leave-one-out sensitivity analysis of the post-hoc single-arm meta-analysis. The magnitude and direction of the anatomical response remained consistent (pooled mean reduction = − 102.05 μm; 95% CI: −145.24 to − 58.86; I2 = 89.5%), supporting the robustness of CMT reduction after surgery in glaucomatous eyes

The pooled pairwise analysis showed no significant difference in CMT reduction between groups, with a mean difference of + 17.59 μm (95% CI: −29.37 to − 64.54; *p* = 0.46; I2 = 81.4%) (Fig. 2A). Given the substantial heterogeneity, we performed an exploratory leave-one-out sensitivity analysis. Excluding the outlier study [2], reduced the heterogeneity (I2 = 0.0%) and showed a higher improvement in CMT in non-glaucomatous eyes (+ 39.50 μm; 95% CI: 11.48.37 to 67.53; I2 = 0.0%) with clearer benefit in this group (Fig. 2C). However, this was treated as an exploratory finding. Important to note, this study [2] included advanced glaucoma, potentially introducing heterogeneity in the retinal structure and postoperative remodeling, which may explain some of the variability between studies.

Interestingly, the post-hoc single-arm analysis demonstrated that glaucomatous eyes still experienced a significant anatomical improvement (− 102.05 μm; 95% CI: −145.24 to − 58.86; I2 = 89.5%) (Fig. 2B). Notably, excluding Ko et al.[2] from the single-arm analysis reduced the magnitude of this effect (− 75.17 μm; 95% CI: −92.29 to − 58.05; I2 = 22.4%) (Fig. 2D).

**Fig. 3:**
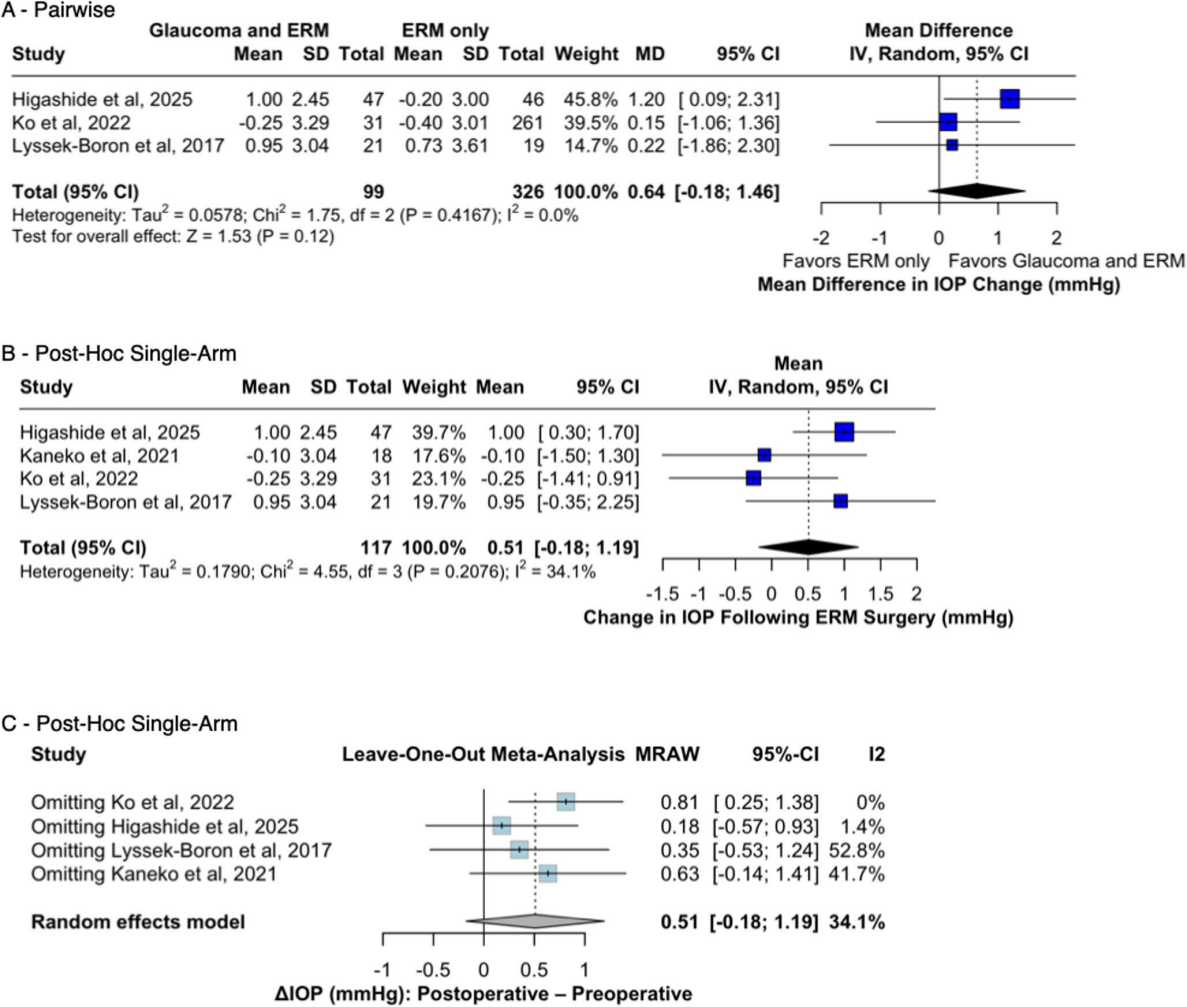
Intraocular pressure (IOP) outcomes – forest plot summarize and sensitivity analyses after vitrectomy for epiretinal membrane (ERM) in glaucomatous eyes. (**A**) Pairwise meta-analysis comparing IOP variation between glaucomatous and non-glaucomatous eyes. No significant difference was found (mean difference = + 0.64 mmHg; 95% CI: −0.18 to 1.46; *p* = 0.12; I2 = 0.0%), suggesting that IOP changes postoperatively were similar between groups. (**B**) Post-hoc single-arm meta-analysis pooling glaucomatous eyes only (*n* = 117). The overall mean IOP change was not statistically significant (mean = + 0.51 mmHg; 95% CI: −0.18 to 1.19; *p* = 0.15; I2 = 34.1%), indicating a neutral effect of ERM surgery on IOP in this population. (**C**) Leave-one-out sensitivity analysis for single-arm meta-analysis evaluating intraocular pressure (IOP) change, demonstrated stability, with no single study significantly altering the overall pooled estimate (+ 0.51 mmHg; 95% CI: − 0.18 to 1.19; *p* = 0.145; I2 = 34.1%)

IOP and glaucoma medication (eye drops). No significant difference in IOP change was observed between glaucomatous and non-glaucomatous eyes in the pairwise meta-analysis (mean difference = + 0.64 mmHg; 95% CI: −0.18 to 1.46; *p* = 0.12; I2 = 0.0%) (Fig. 3A). Similarly, the post-hoc single-arm analysis among glaucomatous eyes revealed no difference in IOP after surgery (+ 0.51 mmHg; 95% CI: −0.18 to 1.19; I2 = 34.1%), suggesting overall IOP stability postoperatively (Fig. 3B).

The post hoc leave-one-out sensitivity analysis showed that no individual study had a substantial impact on the overall ΔIOP estimate (Fig. 3C).

Pairwise meta-analysis of the quantity of glaucoma medication use was not feasible. However, we were able to assess this data through a post-hoc single-arm meta-analysis. We found that patients with glaucoma demonstrated an increase in the number of glaucoma medications postoperatively (mean difference: +0.61 medications; 95% CI: 0.31 to 0.92; I2 = 0.0%) (Supplemental Fig. 1).

Adverse Events.

Vitrectomy was generally safe, with five studies [3, 5, 6, 8, 9] reporting no intraoperative or postoperative complications. No cases of retinal detachment, endophthalmitis, or reintervention were observed. Although some studies noted structural changes, such as reduced ganglion cell layer thickness [6] or theoretical risks related to inner retinal damage [9], these findings were not associated with measurable functional decline or visual acuity loss.

Conversely, four studies reported adverse events with potential clinical impact, such as late visual acuity loss in 17% of glaucomatous eyes [1], postoperative visual acuity worsening (16.1%), increased optic disc cupping (9.7%), and a higher load of antiglaucoma drops [2], cystoid macular edema in 35.3% of glaucomatous and 44.1% of non-glaucomatous eyes [4], and localized visual field sensitivity loss in the glaucomatous hemifield [7]. Reporting was heterogeneous across studies, limiting quantitative synthesis.

### Quality assessment

One study [3] presented a serious risk of bias due to confounding and participant selection, while the remaining seven had a moderate risk, mainly from confounding and participant selection. No study showed a high risk in other domains.

According to the GRADE approach [14], certainty of evidence was high for all primary outcomes (ΔCMT, ΔBCVA, and ΔIOP). Although most studies were observational, no downgrading was applied for risk of bias beyond moderate concerns, as results were consistent across sensitivity analyses. Imprecision was not a concern because confidence of intervals did not cross thresholds of clinical importance. Inconsistency was judged as low, with heterogeneity of 30% for BCVA, 81% for CMT in the primary analysis (reduced to 0% after exclusion of one outlier), and 0% for IOP. Indirectness was not considered serious, as populations, interventions, and outcomes directly addressed the review question (Supplemental Fig. 4).

Narrative outcomes not meta-analyzed.

Although visual field progression, RNFL thickness, and GCC thinning were listed as secondary outcomes in the PROSPERO protocol, these endpoints were not consistently reported across the included studies and could not be pooled. Narrative assessment revealed that visual field decline was noted in two studies [7, 23], mainly localized to the glaucomatous hemifield sensitivity loss after ILM peeling [7], and central-to-nasal deterioration following vitrectomy with ILM peeling [23]. Regarding OCT-based metrics, RNFL and GCC thinning were occasionally described [4, 9], but reporting were heterogeneous (such as segmentation protocols and timepoints), precluding meta-analysis. Together, these reports suggests that postoperative CV deterioration may occur in a subgroup of glaucomatous eyes, particularly those with preexisting defects. However, the magnitude, timing, and determinants of this risk are uncertain due to inconsistent reports and a lack of pooled estimates.

## Discussion

In this systematic review and pairwise meta-analysis of 6 studies and 779 eyes (256 glaucomatous and 523 non-glaucomatous eyes), we found that eyes with glaucoma achieved less visual and anatomical improvement after PPV for idiopathic ERM when compared to non-glaucomatous eyes. However, our post-hoc single-arm meta-analysis of 325 glaucomatous eyes revealed a clinically significant mean gain in visual acuity (10.03 ETDRS letters; 95% CI: 5.18 to 14.87) and a significant anatomical improvement (− 102.05 μm; 95% CI: −145.24 to − 58.86) (Figs. 1 and 2). Importantly, IOP remained stable after surgery (*p* = 0.15) for both groups, although a single-arm analysis revealed an increase in the use of glaucoma drops after surgery (Fig. 3). Evidence certainty was high across all outcomes (GRADE) (Supplemental Fig. 4).

Baseline disease severity is important to consider in all included studies (Table 1). Most of the glaucoma eyes included in this meta-analysis had mild to moderate glaucoma, while advanced disease was poorly reported and mainly concentrated in a single study[2]. In the same way, the staging of ERM was predominantly limited to the initial stages, with few eyes having advanced ERM. As postoperative visual recovery after ERM surgery is influenced by residual inner retinal function, these baseline characteristics may have contributed to the magnitude of visual and anatomical gains observed. Therefore, the benefits are most applicable to patients with mild to moderate glaucoma and early stages of ERM.

Although ERM surgery can provide significant anatomical and functional improvement in eyes with glaucoma, the magnitude of benefit is consistently attenuated compared to non-glaucomatous eyes. Glaucoma severity therefore emerges as an important modifier of postoperative outcomes, as advanced disease implies reduced retinal reserve that may limit functional recovery even when macular anatomy improves. As most of the included studies predominantly involved patients with mild to moderate glaucoma, caution is needed when extrapolating these estimates to advanced glaucoma, in which neurofunctional benefit and vulnerability may differ.

Although VF progression could not be quantitatively synthesized due to heterogeneous and limited extractable data, available evidence suggests that glaucomatous eyes may experience postoperative deterioration of central VF sensitivity [7, 23] despite improvements in visual acuity and stable IOP. Specially, a comparative study [23] showed significant worsening of central (≤ 10°) VF sensitivity exclusively in glaucomatous eyes following vitrectomy with ILM peeling. These changes were predominantly localized to the paracentral regions and were not associated with postoperative IOP elevation, supporting the hypothesis that preexisting retinal cell vulnerability. Importantly, the risk appears heterogeneous and has been reported in eyes with advanced or unstable glaucoma, as well in older patients, systemic hypertension and longer axial length. Together, these findings suggest that ERM surgery in glaucoma, particularly in moderate to advanced disease, may carry a clinically relevant risk of central VF loss that should be considered during surgical planning and patient counseling.

In practice, PPV remains an effective approach for the treatment of ERM. However, functional and anatomical outcomes tend to be less favorable in glaucomatous eyes. These limitations likely reflect preexisting retinal damage, including ganglion cell dysfunction and reduced retinal reserve[2–4, 17, 18, 22. OCT-based biomarkers may help explain the attenuated functional recovery observed in glaucomatous eyes. Ectopic inner foveal layers (EIFL) are recognized negative prognostic marker in idiopathic ERM and are associated with poorer postoperative visual recovery [24]. In the presence of concomitant glaucoma, EIFL may further limit functional gains, by reflecting more advanced damage to the inner retina [17]. However, direct comparative evidence demonstrating a higher prevalence of EIFL in glaucomatous ERM eyes remains limited, and this relationship should be interpreted as a plausible hypothesis rather than a confirmed association.

According to this, Govetto et al. [3], reported that nearly half of the glaucomatous eyes had advanced disease with persistent microcystic changes in the inner nuclear layer, suggesting irreversible retinal degeneration that may prevent anatomical improvement from translating into functional benefit. Similarly, Han et al. [4] attributed modest visual gains to the presence of moderate glaucomatous damage, postoperative cystoid macular edema, and chronic retinal changes, such as thinning of the ganglion cell complex and interdigitation zone disruption. These chronic alterations may limit the potential for visual recovery despite successful anatomical repair [18, 20, 22, 23].

Even in the presence of these structural and functional restrictions, our post hoc analysis revealed that glaucomatous eyes still derive significant benefit from PPV. This finding reinforces the concept that, although the improvements may be less marked, both visual and anatomical outcomes remain clinically relevant.

Notably, Ko et al. [2] emerged as a major contributor to heterogeneity in CMT thickness analysis (Fig. 2). This study predominantly included patients with severe glaucoma, which may partly explain the divergent anatomical response observed and further supports the notion that glaucoma severity influences postoperative outcomes. Greater CMT reduction observed in glaucomatous eyes, possibly reflecting baseline differences, such as a higher (though not statistically significant) CMT in glaucomatous eyes, or retinal thinning due to degeneration rather than true anatomical improvement. Exclusion of this study [2] in an exploratory sensitivity analyses reduced heterogeneity, however, was not used to define the primary conclusions, serving instead to highlight clinical heterogeneity related to disease severity.

These findings underscore the need for randomized controlled trials with careful control of confounders and stratification by glaucoma severity. Most of the included studies focused on mild to moderate cases, leaving a gap in understanding surgical outcomes in advanced glaucoma. A recent systematic review showed that older glaucomatous patients with severe baseline visual field defects are at increased risk of postoperative central-to-nasal field deterioration after ILM peeling [19]. Additional studies have demonstrated that postoperative visual field decline is more likely in eyes with thinner ganglion cell layers, longer axial lengths, and more advanced stages of glaucoma[20, 21].

In addition to visual and anatomical outcomes, postoperative glaucoma management deserves attention. Although IOP remained stable compared to baseline, eyes with glaucoma required a higher number of topical medications after surgery, suggesting that maintaining glaucoma control may become more challenging after vitrectomy.[19 ]Nonetheless, the overall IOP stability reinforces the procedural safety of PPV with ILM peeling in glaucomatous eyes, at least from the standpoint of pressure control. These findings highlight the importance of individualized postoperative glaucoma management and underscore the need for coordinated care with glaucoma specialists when planning PPV in these patients.

Vitrectomy was generally safe, with no serious adverse events reported. Some studies noted late visual acuity loss [1], increased optic disc cupping, greater use of antiglaucoma medications [2], cystoid macular edema [4], or localized visual field loss [7], but none were directly attributable to the procedure.

This study has several limitations. Patients with glaucoma and ERM with microcystic macular edema are predictors of visual acuity decline, and previous studies have shown that PPV may be more beneficial than observation alone [25, 26]. Advanced disc cupping, thinner ganglion cell layer, and preoperative VF deficits may affect the results of treatment of ERM in glaucoma patients [5, 20]. The number of included studies was limited, precluding robust subgroup analyses by glaucoma severity or ERM stage, We also lacked sufficient data to explore the impact of surgical factors, such as dye use or ILM peeling. In addition, we could not test the efficacy and safety of PPV for ERM in more advanced glaucoma stages, since most of the included studies in this analysis focused on mild to moderate glaucoma. Importantly, several secondary outcomes prespecified in PROSPERO, such as VF progression, RNFL thickness, and GCC thinning, could not be quantitatively synthesized due to heterogenous reporting and lack of standardized data. Therefore, should be interpreted as hypothesis-generating. Finally, all included studies were observational, with potential residual confounding. Moreover, important variables such as lens status, timing of cataract surgery, glaucoma type (e.g., primary open-angle glaucoma, normal-tension glaucoma, and pseudoexfoliative glaucoma) were not consistently reported, limiting the possibility of subgroup analyses and adjustment for these confounders.

To test our results and address these limitations, we conducted a series of subgroup and sensitivity analyses. Additionally, we conducted a single-arm post hoc analysis and incorporated meta-regressions where feasible. Moreover, we assessed the overall certainty of evidence using the GRADE [14, 15] approach. Sensitivity and subgroup analysis generally confirmed our overall results, and we found high certainty of evidence for all outcomes.

## Conclusion

PPV with ILM peeling in eyes with glaucoma is associated with clinically meaningful improvements in visual acuity and macular thickness and no consistent change in IOP, although additional glaucoma medications may be required. However, neurofunctional safety, particularly central VF integrity, cannot be guaranteed. Available evidence suggests a small but clinically relevant risk of postoperative central VF deterioration in glaucomatous eyes, especially in patients with moderate-to-advanced disease or additional risk factors. Therefore, these results are most applicable to mild to moderate glaucoma, and carefully patient selection, counseling, and postoperative VF monitoring remain essential.

## Supplementary Information

Supplementary file 1.

Supplementary file 2.

Supplementary file 3.

Supplementary file 4.

Supplementary file 5.

Supplementary file 6.

## Data Availability

All data is within the manuscript.

## Abbreviations

BCVA: Best-corrected visual acuity
CI: Confidence intervals
CMT: Central macular thickness
EIFL: Ectopic inner foveal layers
ERM: epiretinal membrane
ETDRS: Early Treatment Diabetic Retinopathy Study
GCC: ganglion cell complex
GRADE: Grading of Recommendations Assessment, Development and Evaluation
I2: Percentage of total variation across studies due to heterogeneity rather than chance
ILM: Internal limiting membrane
IOP: Intraocular pressure
MRAW: Mean raw difference
PPV: Pars plana vitrectomy
PRISMA: Preferred Reporting Items for Systematic Reviews and Meta-Analyses
R2: Proportion of between-study variance explained by covariates in meta-regression
RNFL: retinal nerve fiber layer
ROBINS-I: Risk of Bias in Non-Randomized Studies of Interventions tool
SD: standard deviation
VF: visual field
