# Supplementary figures and images for: Impact of glaucoma on outcomes after epiretinal membrane surgery. a pairwise and post-hoc single-arm meta-analysis
Source: Graefes Arch Clin Exp Ophthalmol. 2026 Mar 26;264(7):1949–61. doi: 10.1007/s00417-026-07188-2 (PMC13342141; doi:10.1007/s00417-026-07188-2)

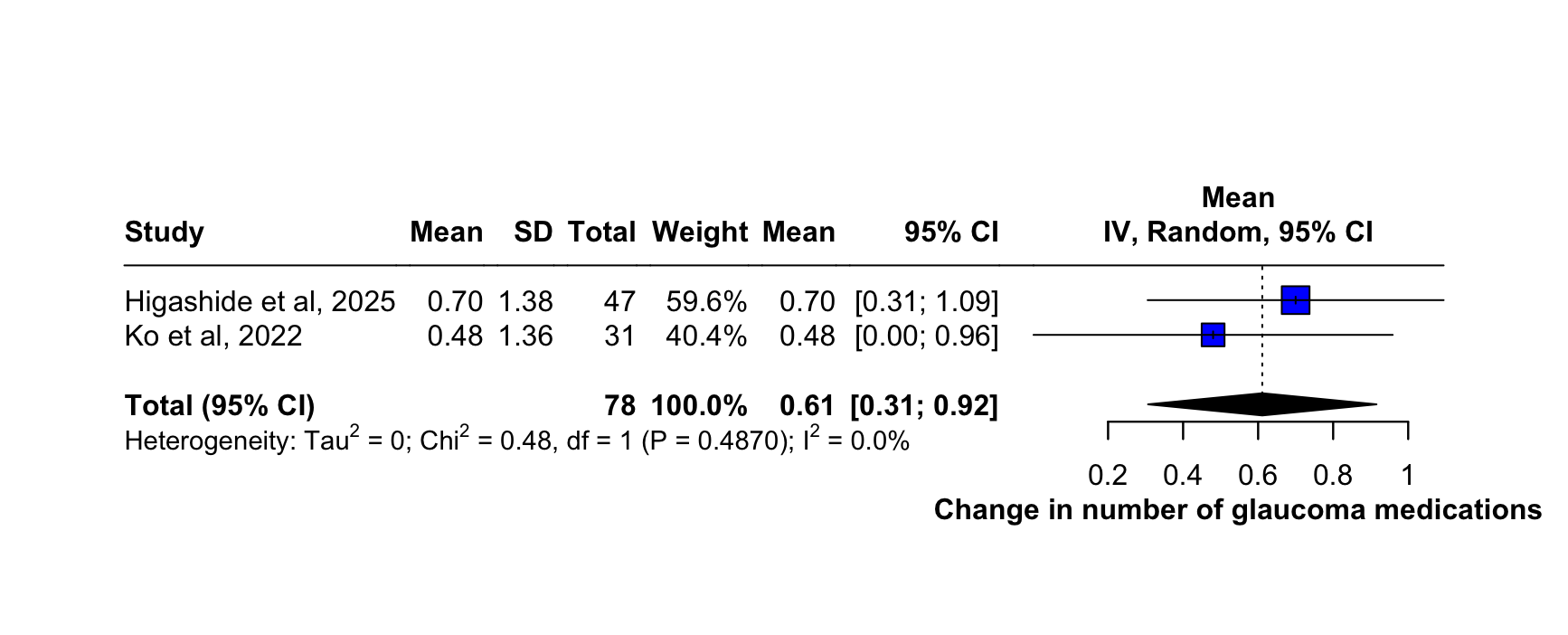

Supplement: Supplementary file 1 [file 417_2026_7188_MOESM1_ESM.tiff]

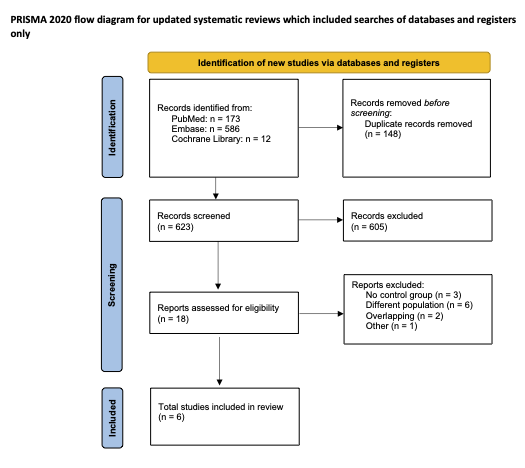

Supplement: Supplementary file 2 [file 417_2026_7188_MOESM2_ESM.tiff]

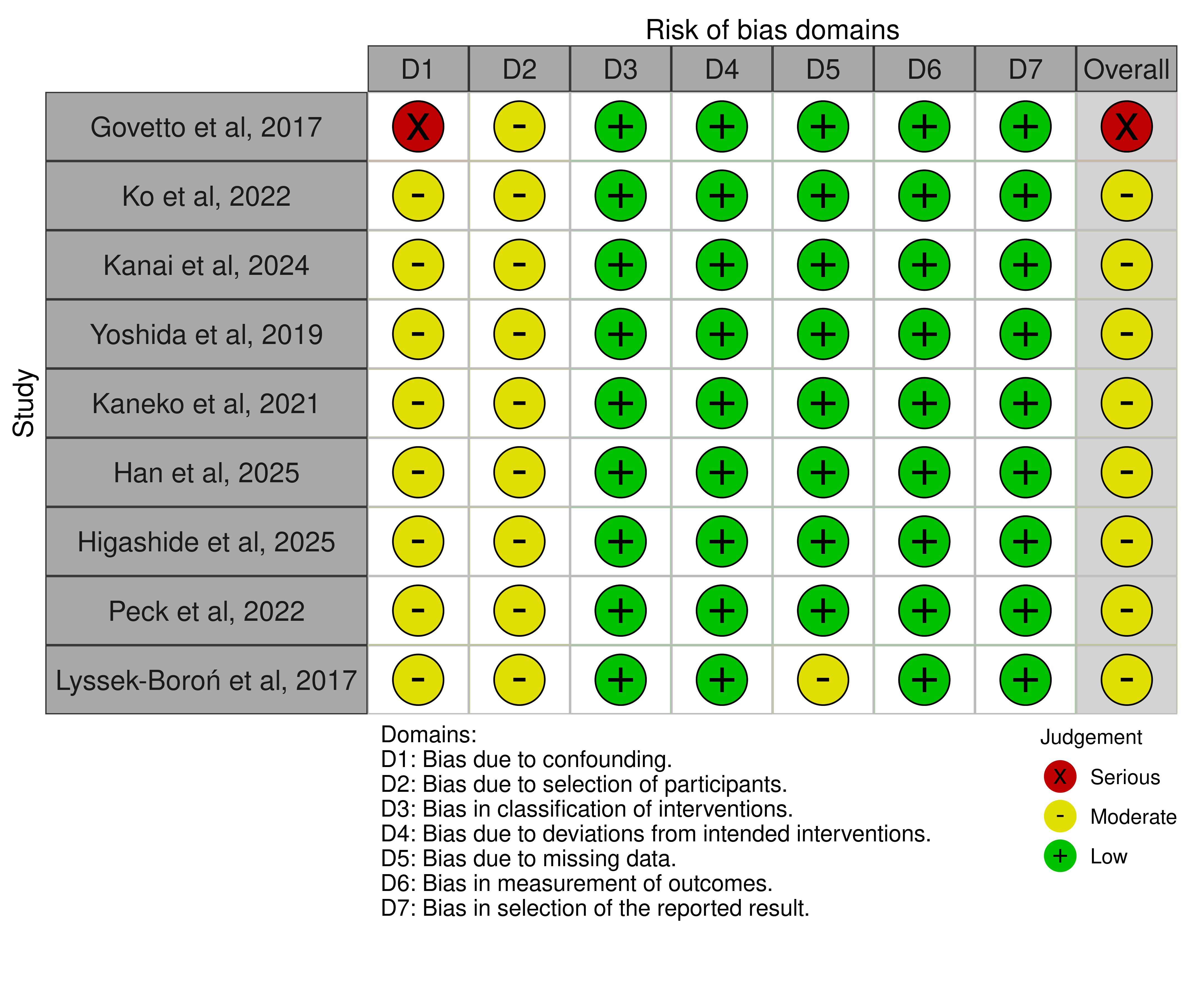

Supplement: Supplementary file 5 [file 417_2026_7188_MOESM5_ESM.tiff]

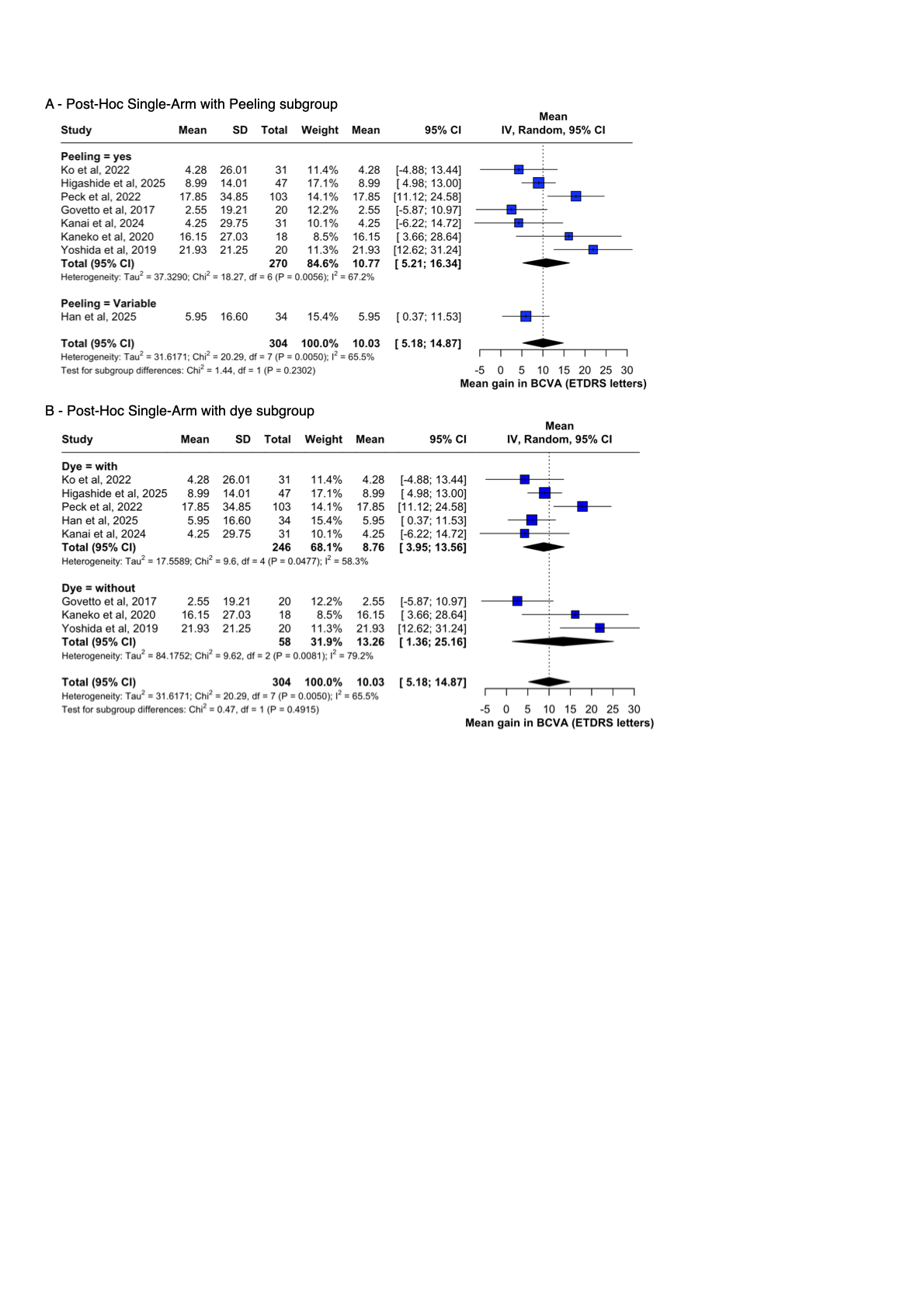

Supplement: Supplementary file 6 [file 417_2026_7188_MOESM6_ESM.tiff]
