# Supplementary material for: Impact of glaucoma on outcomes after epiretinal membrane surgery. a pairwise and post-hoc single-arm meta-analysis
Source: Graefes Arch Clin Exp Ophthalmol. 2026 Mar 26;264(7):1949–61. doi: 10.1007/s00417-026-07188-2 (PMC13342141; doi:10.1007/s00417-026-07188-2)
Supplement: Supplementary file 3 [file 417_2026_7188_MOESM3_ESM.docx]

**Question:** Glaucoma compared to Without Glaucoma in Epiretinal Membrane Surgery

| **Certainty assessment** | | | | | | | **№ of patients** | | **Effect** | **Certainty** | **Importance** |
| --- | --- | --- | --- | --- | --- | --- | --- | --- | --- | --- | --- |
| **№ of studies** | **Study design** | **Risk of bias** | **Inconsistency** | **Indirectness** | **Imprecision** | **Other considerations** | **Glaucoma** | **Without Glaucoma** | **Relative (95% CI)** |  |  |
| **ΔCMT** | | | | | | | | | | | |
| 3 | non-randomised studies | not serious | not serious^a^ | not serious | not serious | none | 85/404 | 319/404 | **Rate ratio 17.59** (-29.37 to 64.54) | ⨁⨁⨁⨁ High^a^ | IMPORTANT |
| **ΔBCVA** | | | | | | | | | | | |
| 6 | non-randomised studies | not serious | not serious | not serious | not serious | none | 235/739 | 504/739 | **Rate ratio -6.95** (-11.54 to -2.35) | ⨁⨁⨁⨁ High | IMPORTANT |
| **ΔIOP** | | | | | | | | | | | |
| 3 | non-randomised studies | not serious | not serious | not serious | not serious | none | 99/425 | 326/425 | **Rate ratio 0.64** (-0.18 to 1.46) | ⨁⨁⨁⨁ High | IMPORTANT |

**CI:** confidence interval

#### Explanations

a. I2 = 65.5%
