# Supplementary material for: Impact of glaucoma on outcomes after epiretinal membrane surgery. a pairwise and post-hoc single-arm meta-analysis
Source: Graefes Arch Clin Exp Ophthalmol. 2026 Mar 26;264(7):1949–61. doi: 10.1007/s00417-026-07188-2 (PMC13342141; doi:10.1007/s00417-026-07188-2)
Supplement: Supplementary file 4 [file 417_2026_7188_MOESM4_ESM.docx]

Search Strategies

April 27, 2025

Pubmed

(glaucoma OR "Ocular Hypertension"[Mesh] OR “ocular hypertension” OR glaucomatous) AND ("Epiretinal Membrane"[Mesh] OR “epiretinal membrane” OR “epimacular membrane” OR “macular pucker”) AND (vitrectomy OR PPV OR MIVS OR peeling OR “ILM removal” OR “ILM delamination”)

Embased

(glaucoma OR "Ocular Hypertension" OR “ocular hypertension” OR glaucomatous)

AND ("Epiretinal Membrane" OR “epiretinal membrane” OR “epimacular membrane”

OR “macular pucker”) AND (vitrectomy OR PPV OR MIVS OR peeling OR “ILM removal” OR “ILM delamination”)

Cochrane Library

(glaucoma OR "Ocular Hypertension" OR “ocular hypertension” OR glaucomatous)

AND ("Epiretinal Membrane" OR “epiretinal membrane” OR “epimacular membrane”

OR “macular pucker”) AND (vitrectomy OR PPV OR MIVS OR peeling OR “ILM removal” OR “ILM delamination”)
